# Identification of Natural Compound Carnosol as a Novel TRPA1 Receptor Agonist

**DOI:** 10.3390/molecules191118733

**Published:** 2014-11-14

**Authors:** Chenxi Zhai, Qing Liu, Yuxin Zhang, Shifeng Wang, Yanling Zhang, Shiyou Li, Yanjiang Qiao

**Affiliations:** 1 Key Laboratory of TCM-information Engineer of State Administration of TCM, School of Chinese Pharmacy, Beijing University of Chinese Medicine, Beijing 100102, China; E-Mails: zhaichenxi1991@163.com (C.Z.); liuqingtcm@gmail.com (Q.L.); zhangyuxinwjzy@163.com (Y.Z.); alana6268@126.com (S.W.); collean_zhang@163.com (Y.Z.); 2 Laboratory of Genome Variations and Precision Bio-Medicine, Beijing Institute of Genomics, Chinese Academy of Sciences, Beijing 100101, China

**Keywords:** TRPA1, canosol, anti-nociceptive, calcium mobilization

## Abstract

The transient receptor potential ankyrin 1 (TRPA1) cation channel is one of the well-known targets for pain therapy. Herbal medicine is a rich source for new drugs and potentially useful therapeutic agents. To discover novel natural TRPA1 agonists, compounds isolated from Chinese herbs were screened using a cell-based calcium mobilization assay. Out of the 158 natural compounds derived from traditional Chinese herbal medicines, carnosol was identified as a novel agonist of TRPA1 with an EC_50_ value of 12.46 µM. And the agonistic effect of carnosol on TRPA1 could be blocked by A-967079, a selective TRPA1 antagonist. Furthermore, the specificity of carnosol was verified as it showed no significant effects on two other typical targets of TRP family member: TRPM8 and TRPV3. Carnosol exhibited anti-inflammatory and anti-nociceptive properties; the activation of TRPA1 might be responsible for the modulation of inflammatory nociceptive transmission. Collectively, our findings indicate that carnosol is a new anti-nociceptive agent targeting TRPA1 that can be used to explore further biological role in pain therapy.

## 1. Introduction

Transient receptor potential cation channel, subfamily A, member 1 (TRPA1), a member of the transient receptor potential (TRP) family, was initially identified in cultured human lung fibroblasts [1]. TRPA1 is found to be primarily expressed in sensory neurons [2,3,4]. It is also widely expressed outside of the nervous system, such as urinary bladder, prostate, cerebral artery and airway [5,6,7,8]. TRPA1 plays a key role in chronic inflammation and hyperalgesia [9,10]. Functional studies of TRPA1 have mainly focused on its role in diverse sensory processes including inflammatory pain and bladder hyperalgesia [11,12]. TRPA1 has also been reviewed as a potential target for cancer, and airway diseases [13,14]. Many kinds of TRPA1 antagonists, such as HC-030031, were selected as candidates to treat hyperalgesia in persistent inflammatory states [15]. TRPA1 antagonist GRC-17536 has already reached clinical Phase 2 stage to cure chronic pain, CB-625 has reached clinical Phase 1 stage to cure surgical pain [16]. Moreover, TRPA1 agonist ASP7663 was reported to have an analgesic effect in animal models of the colorectal distension [17].

TRPA1 is best known as a sensor of pain, cold and environmental irritants. Researchers have found that TRPA1 is sensitive to cold temperatures of less than 17 °C [18], and could be activated by a large variety of agents, especially pungent or irritant compounds. Some of the agents are extracted from herbs, such as allicin from garlic and cinnamaldehyde (CA) from cinnamon [19,20]. A number of natural products targeting TRPA1 were identified during the last decade [16]. The discovery of novel TRPA1 agonist would be helpful for the interpretation of TRPA1 functional mechanism. TRPA1 is a Ca^2+^-permeable, non-selective cation channel [21]. Based on its channel characteristics, a calcium mobilization assay can be used to monitor the activation of TRPA1.

Traditional Chinese medicine (TCM) has been practiced in the Chinese health care system for more than 2000 years. A variety of components derived from TCM have been isolated and identified, providing a good source for drug screening. One hundred and fifty eight compounds isolated from 75 kinds of Chinese herbs were picked for TRPA1 agonist primary screening in this study. These herbs were commonly used for pain therapy in China, such as *Panax ginseng*, *Alpinia katsumadai*, *Lonicera japonica* and so on. Part of the mechanism for pain therapy has been reported in recent years. For example, ginseng total saponins showed antinociceptive activity in rats [22], cardamonin isolated from *Alpinia katsumadai* produced anti-nociceptive effects via blocking expression of cyclooxygenase-2 and transglutaminase-2 [23]. N-butanol fraction prepared from dried flowers of *Lonicera japonica* and dried roots of *Anemarrhena asphodeloides* also displayed analgesic and anti-inflammatory effect* in vivo* [24]. Although the analgesic mechanisms of some Chinese herbs, such as *Fraxinus rhychophylla*, have not been reported yet, the herbal medicines have been used to treat pain for many years in China.

To obtain novel active TRPA1 agonists, a cell-based calcium mobilization screening assay was established to investigate 158 compounds isolated from TCM. Here we report the discovery of carnosol, a potential anti-nociceptive agent, as a novel agonist of TRPA1 via calcium mobilization assay. Furthermore, the stimulating effect of carnosol could be inhibited by a specific TRPA1 inhibitor A-967079.

## 2. Results and Discussion

### 2.1. HEK293/TRPA1 Cell Line Validation

**Figure 1:**
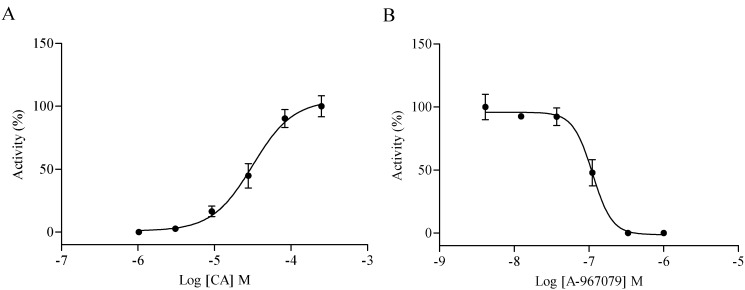
HEK293/transient receptor potential ankyrin 1 (TRPA1) cell line validation. (**A**) Concentration-response curves of cinnamaldehyde (CA). The EC_50_ value of CA was 31.54 µM; (**B**) Concentration-response curves of A-967079 (evoked by 90 µM CA). The IC_50_ value of A-967079 was 0.11 µM.

Cell-based calcium mobilization assay for high-throughput screening (HTS) has been used to study the function of G-protein coupled receptors (GPCR) and Ca^2+^-permeable ion channels by measuring the changes of intracellular free Ca^2+^ levels. To screen TRPA1 agonist through HEK293 cells over-expressing human TRPA1 receptor, the utility of the cell line was first validated using known TRPA1 agonist and antagonist. The EC_50_ value of TRPA1 agonist CA was determined as 31.54 µM (Figure 1A), and the IC_50_ of TRPA1 antagonist A-967079 stimulated with 90 µM CA (EC_80_ value) was determined as 0.11 µM (Figure 1B), which were consistent with previously reported data [25,26].

**Figure 2:**
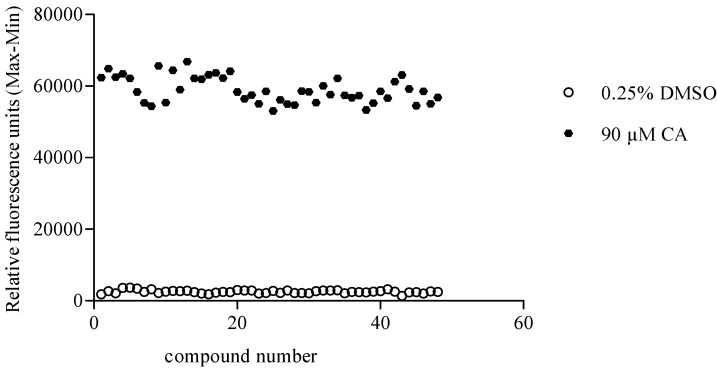
Z' factor plot of screening for TRPA1 agonists using the calcium mobilization assay in 96-well plate. Ninety micromoles of CA and 0.25% DMSO were defined as the positive and negative control, respectively. The Z' value was determined as 0.78.

Subsequently, the robustness of the assay was assessed by determining the Z' factor. Z' factor is used for assessing the quality of HTS assays. In general, a Z' value above 0.5 suggests that a HTS assay is robust enough [27]. As shown in Figure 2, 90 µM CA and 0.25% DMSO were defined as the positive and negative control. The Z' factor was calculated as follows:

(1) $$Z‣=1-3\times \frac{SD_{\text{po}sitive}+SD_{negative}}{M_{\text{po}sitive}-M_{negative}}$$

The TRPA1 agonist screening assay established in HEK293 cells described in this paper displayed a Z' value of 0.78, which suggested that the screening assay is of high-quality nature.

### 2.2. TRPA1 Agonist Primary Screening

**Figure 3:**
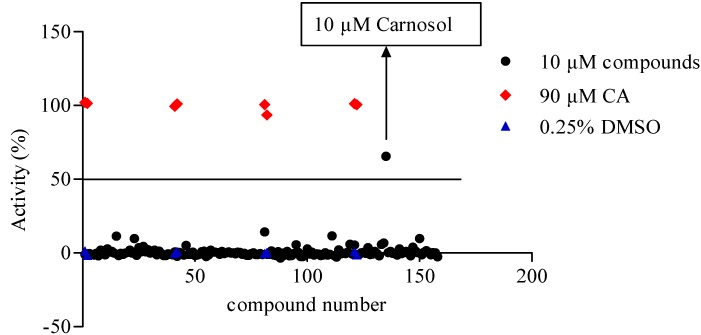
Scatter plot of a collection of 158 Traditional Chinese medicine (TCM) compounds (10 μM). Each dot represents one compound. Ninety μM CA (red dot) and 0.25% DMSO (blue dot) were used as the positive and negative control, respectively. Hits above the cutoff value (50%) were picked for retesting.

We evaluated the activating effect of 158 compounds in HEK293/TRPA1 cells at a concentration of 10 μM using the cell-based calcium mobilization assay (Figure 3), hits above the cutoff value (50%) were picked for retesting. Among all screened compounds, one of them, carnosol, was picked up for further research.

**Figure 4:**
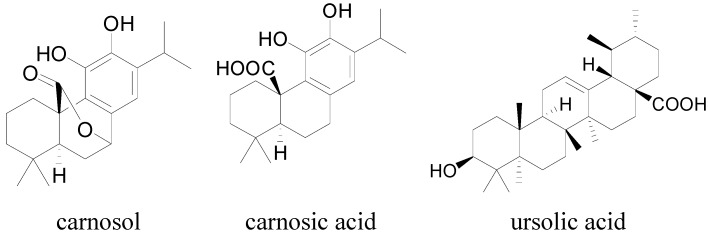
Chemical formula of carnosol, carnosic acid and ursolic acid.

Carnosol is an abundant natural product present in Salvia species, such as *Rosmarinus officinalis* L. (rosemary) and* Salvia officinalis* L. (sage) [28]. *Salvia officinalis* (SO) is a popular species that is grown worldwide due to its culinary, cosmetic and medicinal uses. Some biological activities have been reported for different extracts of SO, such as gastro-protective, anti-oxidative, antibacterial, anti-inflammatory and anti-diabetic effects [29,30,31,32]. An animal study showed that SO extract could be useful in the treatment of chemotherapy-induced peripheral neuropathic [33]. The leaf extracts of SO were proved to process analgesic and anti-inflammatory effects [34]. It was reported that carnosol and ursolic acid inhibited inflammatory phases of formalin test, as well as CA-induced mechanical allodynia [35]. The inhibition of microsomal prostaglandin E2 synthase-1 (mPGES-1) by carnosol and carnosic acid might contribute to the anti-carcinogenic and anti-inflammatory properties of SO [36]. Carnosol, carnosic acid and ursolic acid (Figure 4) showed notable anti-inflammatory and anti-nociceptive effects.

**Figure 5:**
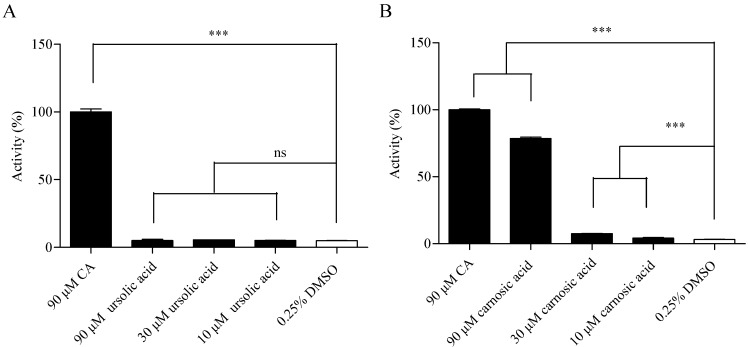
Agonist verification of (**A**) ursolic acid and (**B**) carnosic acid in HEK293/TRPA1 cell line using calcium assay. Ninety micromoles of CA and 0.25% DMSO were used as the positive and negative control respectively. Values are means of replicates of three independent experiments ± standard error. Bars with stars are significantly different from the control group (*** *p* < 0.001).

These three compounds were tested during the primary screening of HEK293/TRPA1, only carnosol showed significant difference compared with the negative control. Since the resemblance of carnosol, ursolic acid and carnosic acid in function and structure characteristics, high concentrations of ursolic acid and carnosic acid were studied to prevent missing positive results in the following research. Ursolic acid caused no significant calcium signal in HEK293/TRPA1 cell line up to 90 µM, while carnosic acid caused calcium signal at 90 µM in HEK293/TRPA1 cell line (Figure 5).

### 2.3. Compound Specificity Determination

False positive result is a major problem for any type of HTS assay. If a compound has the same ability to induce transient calcium influx in HEK293 host cells and HEK293/TRPA1 cells, this compound would be considered as a false positive result. ATP is a well-known intracellular source of energy for all forms of living cells. It elicits intracellular calcium mobilization by activating endogenous P2Y receptors [37], that is the reason why HEK293 cells could be evoked by ATP with significant calcium signal. To exclude false positives, HEK293 host cells were stimulated with tested compounds. Five micromoles of ATP and 0.25% DMSO were used as the positive and negative control, respectively.

**Figure 6:**
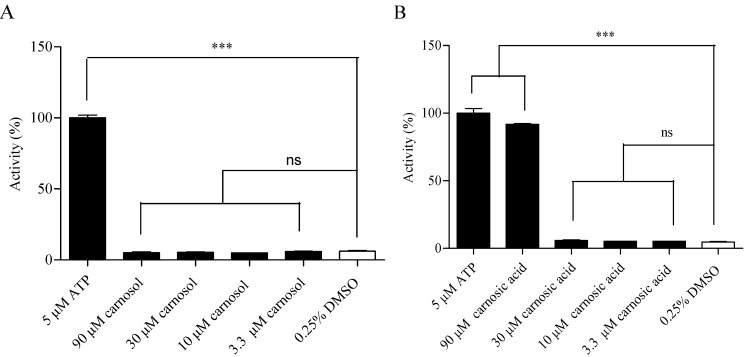
Effect of carnosol (**A**) and carnosic acid (**B**) in HEK293 host cells were evaluated using calcium assay. Five micromoles of ATP and 0.25% DMSO were used as the positive and negative control respectively. Values are means of replicates of three independent experiments ± standard error. Bars with stars are significantly different from the control group (*** *p* < 0.001).

There was no significant difference between carnosol group and 0.25% DMSO group in Figure 6A, suggesting that carnosol-evoked calcium signal was the second messenger signal downstream of TRPA1 receptor. However, carnosic acid acted on HEK293 host cells, indicating that the agonistic action of carnosic acid in HEK293/TRPA1 was false positive result (Figure 6B). The negative reaction of carnosol in HEK293 host cells verified the agonistic action of carnosol on TRPA1 receptor.

### 2.4. Hit Verification

**Figure 7:**
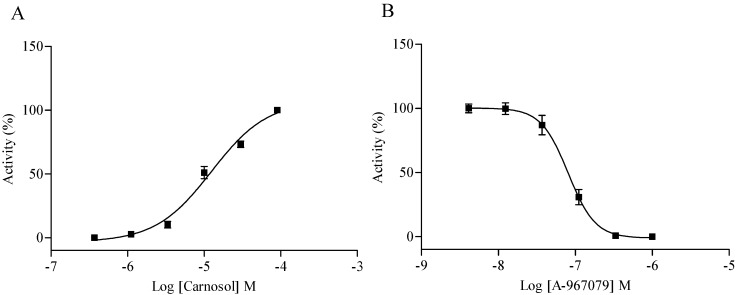
Hit verification in HEK293/TRPA1 cell line. (**A**) Concentration-response curves of carnosol in HEK/TRPA1 cell line. The EC_50_ of carnosol was 12.46 µM; (**B**) Concentration-response curves of A-967079 (evoked by 30 µM carnosol). The IC_50_ value of A-967079 was 80.70 nM.

The activated calcium signal induced by carnosol was comparable to the positive control as showed in Figure 3. To acquire the agonist dose response, we tested the stimulating effect of carnosol at different concentrations in HEK293/TRPA1 cell line. A dose-dependent trend was observed with an EC_50_ value of 12.46 µM (Figure 7A). A-967079 was reported as a potent, highly selective TRPA1 antagonist, which showed >1000-fold selective over other TRP channels, and >150-fold selective over 75 other ion channels, enzymes, and GPCR [26]. A series concentrations of A-967079 were used to block 30 µM carnosol (EC_80_ value) induced calcium mobilization, the IC_50_ value of A-967079 was determined as 80.70 nM (Figure 7B), which further confirmed the agonist effect of carnosol on TRPA1 receptor.

In previous studies, carnosol showed various bio-active functions including anti-tumor, anti-oxidant, anti-angiogenic, anti-platelet, anti-depressant, anti-nociceptive and anti-inflammatory effects [35,38,39,40,41,42]. In this study, carnosol was found to be an agonist of TRPA1 receptor, which probably was responsible for anti-nociceptive and anti-inflammatory effects on animal models [35]. As a TRPA1 agonist, curcumin caused activation and desensitization of TRPA1 channels of multiple mammalian species [43]. The desensitization of TRPA1 by agonists was influenced by calcium-dependent phosphatidylinositol-4,5-bisphosphate (PIP2) pathway, TRPV1, even protons [44]. The agonist-evoked desensitization of TRPA1 might be the reason why carnosol showed anti-nociceptive and anti-inflammatory effects. Still, the precise mechanisms of carnosol analgesic effect remains to be elucidated.

Structurally, TRP channels possess six-transmembrane domain topology. Menthol activation has been reported to be determined by the residues located within S5 (S873V/T874L) [45]. The potencies of the structurally different agonists were assayed in HEK293 cells transiently expressing TRPA1 wild-type and the mutants, the S6 mutations (S943A/I946M) reduced the potencies of all four tested agonists, suggesting that these amino acid residues are in general critical for channel activation [46]. In this study, we demonstrated carnosol as a TRPA1 agonist in HEK293 cells stably expressing human TRPA1, the effects of carnosol on TRPA1 mutants should be studied in further research.

### 2.5. Compound Selectivity Evaluation

**Figure 8:**
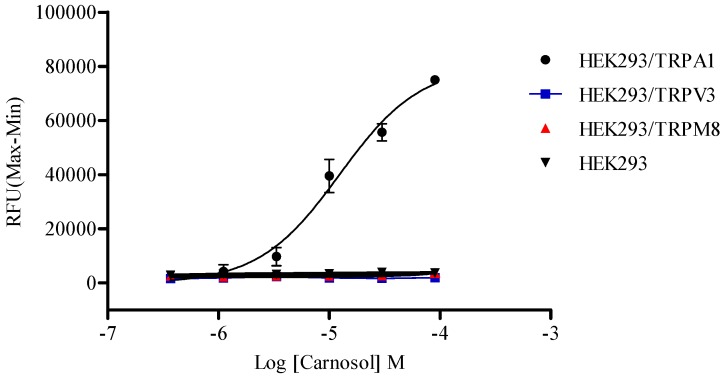
Differential effects of carnosol on TRPA1, TRPM8 and TRPV3 receptors. Serious concentrations of carnosol were tested in host HEK293 and three recombinant cell lines using calcium mobilization assay, respectively.

The TRP channel family is the largest group of receptors that function as targets for analgesics, the heat-sensitive TRPV1, warm-activated TRPV3, cold-responsive TRPA1, and cool-activated TRPM8 channels are especially well studied [47]. To study the specificity of the action target of carnosol, response profiles of HEK293 cells stably expressing TRPM8 and TRPV3, respectively, were detected by carnosol. Icilin and 2-aminoethoxydiphenyl borate (2-APB) were used as agonists to test calcium response in HEK293/TRPM8 and HEK293/TRPV3 cells, respectively. The EC_50_ value of icilin and 2-APB were determined as 0.17 μM and 39.66 μM (data not shown), respectively, which were consistent with reported data [48,49]. Carnosol showed no stimulation signal in HEK/TRPM8 nor in HEK/TRPV3 cell line (Figure 8). Observations made in trigeminal ganglion neurons suggested that eugenol acted on TRPA1 in addition to TRPV1 [50], which might contribute to the analgesic effect of eugenol. We reported here that carnosol had no obvious effect on TRPM8 and TRPV3, whether carnosol can activate TRPV1 remained to be clarified.

### 2.6. Compound Cytotoxicity Evaluation

**Figure 9:**
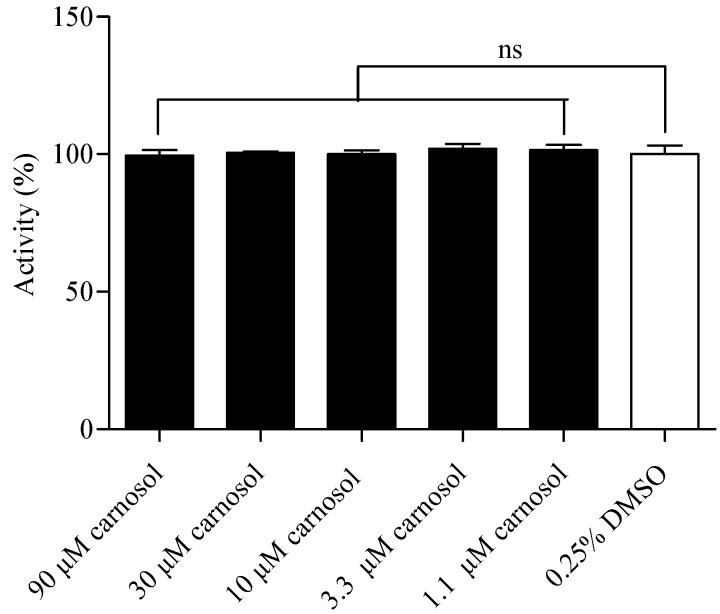
Cytotoxicity evaluation of carnosol in HEK293/TRPA1 cells. The cell viability was determined 2 h after compound stimulation. Values are means of replicates of three independent experiments ± standard error.

A luciferase coupled ATP quantitation assay was used to determine the cytotoxicity of canosol. Compounds were incubated in HEK293/TRPA1 cells for 2 h before luminescence signal measurement. Compared to the control group, canosol showed no significant cytotoxicity on HEK293/TRPA1 cells (Figure 9).

## 3. Experimental Section

### 3.1. Materials

One hundred and fifty eight compounds and CA were purchased from National Institutes for Food and Drug Control (Peking, China) with purities greater than 98%. A-967079, trypsin, ATP, hygromycin B, probenecid and acid red 1 (purity ≥ 98%) were all obtained from Sigma-Aldrich (St. Louis, MO, USA). Fluo-4 AM was purchased from Molecular Probes (Grand Island, NY, USA). Matrigel was purchased from Becton Dickinson (New York, NY, USA). Dulbecco’s modified eagles medium (DMEM) and fetal bovine serum (FBS) were obtained from Gibco BRL (Grand Island, NY, USA). CellTiter-Glo Luminescent Cell Viability Assay kit was purchased from Promega (Madison, WI, USA).

### 3.2. Compounds Preparation

TCM compounds were dissolved in DMSO at 36 mM. For primary screening, TCM compounds were diluted to 4 mM in DMSO. CA and A-967079 were stored at a concentration of 100 mM and 4 mM, respectively. Initial serial dilutions were made in DMSO with compound concentrations at 400× final for concentration-response determinations. For the compound plates, 2 µL of the 400× DMSO solution was added to 160 µL HBSS (5× solution, DMSO concentration 1.25%). The final DMSO concentration in each well was controlled as 0.25% for all the tested compounds.

### 3.3. Cell Culture

HEK 293 cell lines stably expressing human TRPA1, TRPM8 and TRPV3 channels, respectively, were established by HD Bioscience Co. Ltd (SH, China) according to the cell line generation procedure in published articles of our laboratory [51,52]. All HEK293 cell lines used in this study were routinely maintained in DMEM containing 10% FBS, 100 U/mL penicillin, and 100 μg/mL streptomycin in a humidified atmosphere of 5% CO_2_ at 37 °C. HEK293/TRPA1, HEK293/TRPM8 and HEK293/TRPV3 cells were incubated with complete culture medium along with 50 μg/mL hygromycin B.

### 3.4. Agonist Screening Assay

HEK293/TRPA1 cells were seeded at a density of 3.5 × 10^4^ per well into 96-well clear-bottom black plates coated with matrigel and incubated in 5% CO_2_ at 37 °C overnight. On the day of assay, the growth medium was replaced by 100 µL loading buffer containing a final concentration of 4 μM Ca^2+^-sensitive dye Fluo-4 AM and 2 mM acid red 1 in HBSS. The plate was then incubated at 37 °C in dark for 30 min before calcium signal read out. For antagonists study, 80 µL loading buffer was added into each well and 20 µL HBSS containing tested compound was added 10 min prior to calcium-flux measurement.

Cells were transferred to a Flexstation II (Molecular Devices) for experimentation. Basal fluorescence was recorded for 16 s before agonist application. The integrated Flexstation II fluidics system added 25 µL compound (5× solution) from the agonist compound plate to the assay plate containing 100 µL loading buffer solution. Relative fluorescence units (RFU) were read by FlexStation II at 37 °C with an excitation wavelength of 485 nm and an emission wavelength of 525 nm. The fluorescence intensity was read every 1.52 s for 80 s.

Ninety µM CA and 0.25% DMSO were used as the positive and negative control respectively during primary screening of TRPA1 agonist. Hits above the cutoff value (50%) were picked for retesting, percentage of increased calcium signal was calculated as following equation:

(2) $$Activity\%=\frac{RFU_{compound}-RFU_{negative}}{RFU_{positive}-RFU_{negative}}\times 100$$

### 3.5. Compound Specificity Assay

To observe whether the identified compounds induce calcium influx in HEK293 host cells, the cells were treated with various concentrations of tested compounds. Meanwhile, 5 μM ATP and 0.25% DMSO were used as the positive and negative control, respectively. The regulatory activity of tested samples was defined as follows:

(3) $$Activity\%=RFU_{compound}/RFU_{positive}\times 100$$

### 3.6. Compound Selectivity Assay

To study the specificity of the action target of identified compounds, the activating effect on TRPM8 and TRPV3 was tested. HEK293 cell lines stably expressing TRPM8 and TRPV3 receptors were separately stimulated by a serious concentrations of identified compounds using calcium mobilization assay as described above.

### 3.7. Compound Cytotoxicity Assay

HEK293/TRPA1 cells were seeded at 3.5 × 10^4^ per well into 96-well clear-bottom black plates and incubated in 5% CO_2_ at 37 °C overnight. Different concentrations of the compound were added into the 96-well plates and incubated in 5% CO_2_ at 37 °C for 2 h. Luminescence was read by Envision 2100 multilabel reader to detect cells’ viability following incubation with CellTiter-Glo reagent for 10 min.

### 3.8. Statistics

The EC_50_ and IC_50_ values were determined with normalized RFUs using GraphPad Prism 5 software. Data point represents mean ± SEM. The data were analyzed using one way analysis of variance (ANOVA) followed by Dunnett’s multiple comparison test to analyze the differences between group means, *p* value < 0.05 was considered significant.

## 4. Conclusions

In summary, we have successfully developed and evaluated a calcium mobilization assay which were applicable for the screening of TRPA1 agonists. The identified hit carnosol could specifically activate TRPA1 receptor, which could be blocked by a selective TRPA1 antagonist A-967079. Besides, carnosol could not act on HEK293 host cells or other TRPs we tested. Taken together, these results indicate that carnosol, a potential anti-nociceptive agent, was identified as a novel natural TRPA1 agonist, which might play a role of analgesia through activating TRPA1. Thus, carnosol could be a promising drug candidate for pain therapy.

## Supplementary Materials

Supplementary materials can be accessed at: http://www.mdpi.com/1420-3049/19/11/18733/s1.

## Supplementary Files

Supplementary File 1
