# Supplementary material for: Identification of Natural Compound Carnosol as a Novel TRPA1 Receptor Agonist
Source: Molecules. 2014 Nov 14;19(11):18733–46. doi: 10.3390/molecules191118733 (PMC6271858; doi:10.3390/molecules191118733)
Supplement: Supplementary File 1 [file molecules-19-18733-s001.pdf]

Supplementary Materials

Table S1. Compound information and their medicinal plants.

No.	Constituent	PubChem CID	Medicinal Plant
1	Catechin	9064	*Acacia catehu* (L.f.) Willd.
2	Epicatechin	72,276	*Acacia catehu* (L.f.) Willd.
3	sesamin	72,307	*Acanthopanax gracilistylus* W. W. Smith
4	Eleutheroside E	3,084,742	*Acanthopanax senticosus* (Rupr.et Maxim.) Harms
5	Ciwujianoside B	71,571,445	*Acanthopanax senticosus* (Rupr.et Maxim.) Harms
6	hypaconitine	23,337	*Aconitum carmichaeli* Debx.
7	Mesaconitine	416,228	*Aconitum carmichaeli* Debx.
8	Lappaconitine	3886	*Aconitum carmichaelii* Debx.
9	8-*O*-Acetylharpagide	5,459,146	*Ajuga ciliata* Bunge.
10	Calceolarioside B	5,273,567	*Akebia quinata* (Thunb.) Decne.
11	Cardamonin	641,785	*Alpinia katsumadai* Hayata
12	Andrographolide	5,318,517	*Andrographis paniculata* (Burm.f.) Nees
13	mangiferin	5,281,647	*Anemarrhena asphodeloides* Bge.
14	Isoimperatorin	68,081	*Angelica dahurica* (Fisch. Ex Hoffm) Benth. et Hook.f
15	Imperatorin	10,212	*Angelica dahurica* (Fisch. Ex Hoffm) Benth. et Hook.f
16	Byakangelicin	10,211	*Angelica sinensis* (Oliv.) Diels
17	Columbianadin	6,436,246	*Angelica sinensis* (Oliv.) Diels
18	Succinic acid	1110	*Angelica sinensis* (Oliv.) Diels
19	Notoginsenoside R1	441,934	*Angelica sinensis* (Oliv.) Diels
20	Arctigenin	64,981	*Arctium lappa* L.
21	Arctiin	100,528	*Arctium lappa* L.
22	Aristolochic Acid I	2236	*Aristolochia manshuriensis* Kom.
23	Artemisinin	2240	*Artemisia annua* L.
24	Astragaloside	45,006,101	*Astragalus membranaceus* (Fisch.) Bge
25	Folic acid	6037	*Astragalus membranaceus* (Fisch.) Bge
26	Betaine	247	*Astragalus membranaceus* (Fisch.) Bge
27	Atractylenolide I	5,321,018	*Atractylodes macrocephala* Koidz.
28	Atractylenolide III	155,948	*Atractylodes macrocephala* Koidz.
29	Atractylenolide II	14,448,070	*Atractylodes macrocephala* Koidz.
30	Oleuropein	5,281,544	*Canavium album* Raeuseh
31	Hydroxysafflor yellow A	6,443,665	*Carthamus tinctorius* L.
32	Crocin	5,281,233	*Carthamus tinctorius* L.
33	Tracheloside	169,511	*Carthamus tinctorius* L.
34	asiaticoside	108,062	*Centella asiatica* (L.)Urb.
35	Isoferulic acid	736,186	*Cimicifuga heracleifolia* Kom.
36	Neohesperidin	442,439	*Citrus aurantium* L.
37	Nobiletin	72,344	*Citrus aurantium* L.
38	Limonin	179,651	*Citrus aurantium* L.
39	Rutin	5,280,805	*Citrus aurantium* L.
40	Hesperidin	10,621	*Citrus aurantium* L.
41	naringin	442,428	*Citrus aurantium* L.
42	Coptisine	72,322	*Coptis chinensis* Franch.
43	Berbamine dihydrochloride	56,845,155	*Coptis chinensis* Franch.

Table S1. *Cont.*

No.	Constituent	PubChem CID	Medicinal Plant
44	berbamine	10,170	*Coptis chinensis* Franch.
45	Loganin	87,691	*Cornus officinalis* Sieb. et Zucc.
46	Tetrahydropalmatine	72,301	*Corydalis yanhusuo* W. T. Wang
47	Hyperoside	5,281,643	*Crataegus pinnatifida* Bge.
48	Protopine	4970	*Daphne genkwa* Sieb. et Zucc.
49	Genkwanin	5,281,617	*Daphne genkwa* Sieb. et Zucc.
50	Ephedrine Hydrochloride	65,326	*Ephedra sinica* Stapf
51	Icariin	5,471,129	*Epimedium brevicornu* Maxim.
52	Chlorogenic acid	1,794,427	*Eucommia ulmoides* Oliv.
53	Eugenol	3314	*Eugenia caryophllata* Thunb.
54	Phillyrin	44,584,288	*Forsythia suspensa* (Thunb.) Vahl
55	Forsythoside B	44,429,859	*Forsythia suspensa* (Thunb.) Vahl
56	Esculin hydrate	44,134,702	*Fraxinus rhychophylla* Hance
57	fraxetin	5,273,569	*Fraxinus rhychophylla* Hance
58	Peimine	131,900	*Fritillaria cirrhosa* D. Don
59	Peiminine	167,691	*Fritillaria cirrhosa* D. Don
60	Sipeimine	442,977	*Fritillaria cirrhosa* D. Don
61	Gardenoside	442,423	*Gardenia jasminoides* Ellis
62	Genipin	442,424	*Gardenia jasminoides* Ellis
63	Geniposidic acid	443,354	*Gardenia jasminoides* Ellis
64	Gentiobiose	441,422	*Gentiana manshurica* Kitag.
65	gentiopicroside	88,708	*Gentiana manshurica* Kitag.
66	Ginkgolide B	65,243	*Ginkgo biloba* L.
67	Ginkgolide C	16,211,418	*Ginkgo biloba* L.
68	Ginkgolide A	9,909,368	*Ginkgo biloba* L.
69	Daidzin	107,971	*Glycine max* (L.)Merr.
70	Genistin	5,281,377	*Glycine max* (L.)Merr.
71	Liquiritin	503,737	*Glycyrrhiza uralensis* Fisch.
72	Isoliquiritin	5,318,591	*Glycyrrhiza uralensis* Fisch.
73	Liquiritigenin	114,829	*Glycyrrhiza uralensis* Fisch.
74	Glycyrrhizic acid	656,656	*Glycyrrhiza uralensis* Fisch.
75	glycyrrhetic acid	10,114	*Glycyrrhiza uralensis* Fisch.
76	Monoammonium glycyrrhizinate	62,074	*Glycyrrhiza uralensis* Fisch.
77	Narcissoside	5,481,663	*Hedyotis chrysotricha* (Palib.) Merr.
78	Isorhamnetin	5,281,654	*Hippophae rhamnoides* L.
79	Allantoin	204	*Hordeum vulgare* L.
80	colchicine	6167	*Iphigenia indica* A. Gray
81	Ligustrazine HCl	156,709	*Ligusticum chuanxiong* Hort.
82	Senkyunolide A	3,085,257	*Ligusticum chuanxiong* Hort.
83	ferulic acid	445,858	*Ligusticum chuanxiong* Hort.
84	neochlorogenic acid	5,280,633	*Lonicera japonica* Thunb.
85	Isochlorogenic acid A	6,474,310	*Lonicera japonica* Thunb.

Table S1. *Cont.*

No.	Constituent	PubChem CID	Medicinal Plant
86	Luteoloside	5,280,637	*Lonicera japonica* Thunb.
87	Isochlorogenic acid C	6,474,309	*Lonicera japonica* Thunb.
88	3,4-Dicaffeoylquinic acid	5,281,780	*Lonicera japonica* Thunb.
89	Cryptochlorogenic acid	9,798,666	*Lonicera japonica* Thunb.
90	Tormentic acid	73,193	*Mosla chinesis* Maxim.
91	Nardosinone	168,136	*Nardostachys chinensis* Batal.
92	Neferine	159,654	*Nelumbo nucifera* Gaertn.
93	Liensinine	160,644	*Nelumbo nucifera* Gaertn.
94	Isoliensinine	5,274,591	*Nelumbo nucifera* Gaertn.
95	Liensinine perchlorate	71,307,566	*Nelumbo nucifera* Gaertn.
96	Ruscogenin	441,893	*Ophiopogon japonicus* (Thunb.) Ker-Gawl.
97	Paeoniflorin	442,534	*Paeonia lactiflora* Pall.
98	Paeonolum	11,092	*Paeonia suffruticosa* Andr.
99	Protopanaxadiol	11,213,350	*Panax ginseng* C. A. Mey.
100	Protopanaxatriol	22,392,424	*Panax ginseng* C. A. Mey.
101	Ginsenoside Rb1	9,898,279	*Panax ginseng* C. A. Mey.
102	Ginsenoside Rg1	441,923	*Panax ginseng* C. A. Mey.
103	Ginsenoside Re	73,149	*Panax ginseng* C. A. Mey.
104	Ginsenoside Rg2	6,441,009	*Panax ginseng* C. A. Mey.
105	Ginsenoside Rh2	119,307	*Panax ginseng* C. A. Mey.
106	beta-D-Glucopyranoside	3,811,495	*Panax ginseng* C. A. Mey.
107	Panaxadiol	328,778	*Panax ginseng* C. A. Mey.
108	panaxotriol	93,484	*Panax ginseng* C. A. Mey.
109	Perillene	9142	*Perilla frutescens* (L.) Britt.
110	Patchouli alcohol	521,903	*Pogostemon cablin* (Blanco) Benth.
111	Senegenin	200,662	*Polygala tenuifolia* Willd.
112	Amygdalin	656,516	*Prunus armeniaca* L. var. ansu Maxim.
113	Angelicin	10,658	*Psoralea corylifolia* L.
114	Puerarin	5,281,807	*Pueraria lobata* (Willd.) Ohwi
115	Catalpol	91,520	*Rehmannia glutinosa* Libosch.
116	Polydatin	5,281,718	*Reynoutria japonica* Houtt.
117	Aloe emodin	10,207	*Rheum palmatum* L.
118	Sennoside A	73,111	*Rheum palmatum* L.
119	Sennoside B	91,440	*Rheum palmatum* L.
120	Physcion	10,639	*Rheum palmatum* L.
121	Emodin	3220	*Rheum palmatum* L.
122	Chrysophanol	10,208	*Rheum palmatum* L.
123	Pyrogallic Acid	1057	*Rhus chinensis* Mill.
124	Purpurin	6683	Rubia cordifolia L.
125	Protocatechuic acid	72	*Salvia miltiorrhiza* Bge.
126	Tanshinone ⅡA	164,676	*Salvia miltiorrhiza* Bge.
127	Lithospermic acid	6,441,498	*Salvia miltiorrhiza* Bge.
128	Caffeic acid	689,043	*Salvia miltiorrhiza* Bge.

Table S1. *Cont.*

No.	Constituent	PubChem CID	Medicinal Plant
129	Salvianolic acid A	5,281,793	*Salvia miltiorrhiza* Bge.
130	Salvianolic acid B	6,441,188	*Salvia miltiorrhiza* Bge.
131	Tanshinone I	114,917	*Salvia miltiorrhiza* Bge.
132	Cryptotanshinone	160,254	*Salvia miltiorrhiza* Bge.
133	Carnosol	2579	*Salvia officinalis* L.
134	Ursolic acid	64,945	*Salvia officinalis* L.
135	Carnosic acid	65,126	*Salvia officinalis* L.
136	Schisantherin A	151,529	*Schisandra chinensis* (Turcz.)
137	Schizandrin B	108,130	*Schisandra chinensis* (Turcz.)
138	Schisandrin	3,001,664	*Schisandra chinensis* (Turcz.)
139	schisandrin C	119,112	*Schisandra chinensis* (Turcz.)
140	Schizandrol B	68,781	*Schisandra chinensis* (Turcz.)
141	Baicalin	64,982	*Scutellaria baicalensis* Georgi
142	Wogonin	5,281,703	*Scutellaria baicalensis* Georgi
143	scutellarin	185,617	*Scutellaria baicalensis* Georgi
144	Sesamolin	101,746	*Sesamum indicum* L.
145	Sinomenine	5,459,308	*Sinomenium acutum* (Thunb.) Rehd. Et Wils.
146	Astilbin	119,258	*Smilax glabra* Roxb.
147	quercetin-3-rhamnoside	5,280,459	*Sophora japonica* L.
148	Tetrandrine	73,078	*Stephania tetrandra* S. Moore
149	Fangchinoline	73,481	*Stephania tetrandra* S. Moore
150	Triptolide	107,985	*Tripterygium wilfordii* Hook. f.
151	Dchydrodiisoeugenol	5,379,033	*Vaccaria segetalis* (Neck.) Garcke.
152	6-Gingerol	442,793	*Zingiber officinale* Rosc.
153	Bisdemethoxycurcumin	5,315,472	*Zingiber officinale* Rosc.
154	curcumenol	387,977	*Zingiber officinale* Rosc.
155	Borneol	64,685	*Zingiber officinale* Rosc.
156	Betulinic acid	64,971	*Ziziphus jujuba* Mill.var.*spinosa* (Bunge) Hu ex H.F.Chow
157	Betulin	72,326	*Ziziphus jujuba* Mill.var.*spinosa* (Bunge) Hu ex H.F.Chow
158	Jujuboside A	171,446	*Ziziphus jujuba* Mill.var.*spinosa* (Bunge) Hu ex H.F.Chow
